# Remodeling of the *Platynereis* Musculature during Sexual Maturation

**DOI:** 10.3390/biology12020254

**Published:** 2023-02-06

**Authors:** Ina Dahlitz, Adriaan Dorresteijn, Anne Holz

**Affiliations:** Justus-Liebig-Universitaet Giessen, Fachbereich 08 Biologie und Chemie, Institut fuer Allgemeine Zoologie und Entwicklungsbiologie, Heinrich-Buff-Ring 38, 35392 Giessen, Germany

**Keywords:** atrophy, blood vessel proliferation, eleocytes, histolysis, muscle remodeling, sarcolytes

## Abstract

**Simple Summary:**

The internal organ transformations associated with sexual maturity in the polychaete worm *Platynereis dumerilii* have not been systematically analyzed so far. In this study, we focused on the morphology of the longitudinal and transversal muscles in both anterior and posterior regions of the body to understand their transformation during maturation. We found that the longitudinal muscles were smaller and less dense after maturation and were strongly degraded. Moreover, we observed a profound remodeling of the transversal muscles in the posterior segments of both sexes, resulting in strong thickening. Accordingly, the entire musculature in the posterior segments was severely swollen. In addition, we discovered an enormous number of small, blind-ended blood vessels that completely penetrate the musculature in the posterior segments. The different degree of muscle remodeling between anterior and posterior segments reflects the specific tasks of the different body regions after sexual maturation. The anterior segments contain the gametes and thus the degradation of the muscles makes room for the germ cells. By contrast, the swollen muscles and the new blood vessels in the posterior segments coincide with the remodeling of the appendages in this body region into paddle-shaped swimming organs, allowing rapid swimming during nuptial dance and gamete release.

**Abstract:**

Background: The external transformations associated with sexual maturation in *Platynereis dumerilii* (Audouin and Milne Edwards) are well studied, whereas the internal changes along the body axis have not been systematically analyzed. Therefore, we examined muscle morphology in body regions located anterior or posterior to the prospective atokous/epitokous border to generate a structural basis for internal transformations. Results: All dorsal and ventral longitudinal muscles were significantly reduced in size and density after sexual maturation and strongly atrophied, with the greatest decrease in the anterior segments of females. Despite the general reduction in size throughout the longitudinal muscles, we found a specific degradation mechanism for the posterior segments, which were characterized by the formation of secondary bundle-like fibrous structures. In addition, we observed a profound remodeling of the transversal muscles in the posterior segments of both sexes, apparently resulting in excessive thickening of these muscles. Accordingly, the entire transversal muscle complex was severely swollen and ultrastructurally characterized by a greatly increased number of mitochondria. As a possible trigger for this remodeling, we discovered an enormous number of small, blind-ending blood vessels that completely penetrated the longitudinal and transversal muscles in posterior segments. In addition, both the number of visceral muscles as well as their coelothelial covering were reduced during sexual maturation. Conclusions: We hypothesize that a possible reason for the secondary bundling of the longitudinal fibers, as well as the difference in size of the posterior transversal muscles, could be the high degree of posterior vascularization. The different degree of muscle remodeling thus depends on segmental affiliation and reflects the tasks in the motility of the different body regions after maturation. The strongest atrophy was found in the anterior segments, while signs of redifferentiation were encountered in posterior segments, supported by the vigorous growth of vessels supplying the transformed epitokous parapodia and associated muscles, which allows rapid swimming during swarming and gamete release.

## 1. Introduction

Due to the production of many offspring in a short time under laboratory conditions, *Platynereis dumerilli* (Audouin and Milne Edwards) represents a well-studied annelid model organism of Lophotrochozoa [1]. The small size of the oocytes and a transparent egg jelly allows the study of all stages of its embryonic development and derived body structures in detail. The embryonic development, with its typical spiral cleavage, leads through the trochophore stage to the three segmented nectochaete larva. This marine polychaete shows a complex life cycle, adapting to different habitats during the four different life phases (planktonic, benthic, silk tube dwelling, and pelagic stage; [1]), the transitions of which are characterized by metamorphic changes. At the late nectochaete stage, the transition from the pelagic to the benthic way of life is initiated by the first settlement metamorphosis. The second, cephalic metamorphosis, completes the construction of the body plan of the atokous worm shortly afterwards. Further body growth occurs by adding numerous segments in the posterior proliferation zone [1,2,3]. As soon as the atokous worm reaches 70–90 segments or an age of 3–18 months, the final sex-dependent, epitokous metamorphosis (hereafter referred to as sexual maturation) to the mature sex form of the Heteronereis takes place. Morphological changes associated with the swarming ability of both sexes include altered parapodia, atrophy of the intestine, and histolysis of the body wall with an associated reorganization of its muscles. In addition, the body is converted into so-called anterior atokous and posterior epitokous regions, which are separated by an externally visible border [4,5,6]. With the onset of sexual maturation, the eyes enlarge and the number of chromophores on the dorsal surface of the body decreases. The most noticeable change is the cessation of food intake. As the germ cells develop, the females appear completely yellowish due to the accumulation of yolk protein in the oocytes, while the males become whitish in the atokous anterior segments and reddish in the epitokous posterior segments, indicating the growth of the vascular system to improve swimming ability during spawning. *P. dumerilii*, as a semelparous organism, dies after spawning and cannot return to the atokous form [1,2,3,7,8,9,10,11,12].

The first signs of myogenic differentiation appear in *Platynereis* after 28 h with the development of dorsal and ventral longitudinal muscles, followed by the oblique and parapodial muscles. Basic myogenesis is completed when the settlement metamorphosis is finished, but is it required again in each newly built segment [1,13,14]. The polychaete muscles consist of the outer circular and inner longitudinal muscle fibers of the body wall, of diagonal, oblique and parapodial fibers, as well as the muscles of the mesenteries, dissepiments, and the visceral muscles [15]. In Nereidae, the dorsal longitudinal muscle ligaments extend over the entire length of the segment and are regularly attached to the integument. Each lamellar muscle cell of a longitudinal muscle ligament adheres to the basement membrane (BM, a specialized form of the extracellular matrix, ECM) over its entire length. The ventral longitudinal muscle ligaments, with their corresponding BM, are folded over in a double layer [15,16,17,18,19,20,21,22]. The specialized parapodial muscles are attached to BMs at both ends and are made up of fibers that freely run between the ventral nerve cord of the medial body wall on the one hand and the lateral body wall or parapodia on the other. Further muscles extend from the anterior to the posterior wall of each parapodium [2,15,23,24]. Another population of mesodermal cells ventrally differentiates into a specialized longitudinal muscle, the axochord, which is positioned between the ventral nerve cord and the ventral blood vessel [25,26]. All *Platynereis* somatic muscle fibers are mononuclear and display a prominent oblique striation, while the visceral muscles surrounding the midgut consist of longitudinal and circular smooth muscle fibers [16,27,28,29]. 

In addition to the external changes during sexual maturation, there is also an internal restructuring of the somatic muscles. A general remodeling of the posteriorly located longitudinal and transversal muscles is described as one of the prerequisites for swarming behavior in *P. dumerilii* [2,3,6,27]. Muscle remodeling is accompanied by a loss of muscle mass, termed atrophy, with the resulting muscle fragments, the sarcolytes, being released into the body cavity and histolyzed by invading eleocytes. The phagocytosis of sarcolytes is presumably used to generate energy and a turnover of metabolites, since the animals stop feeding at the beginning of maturation and rely on their body reserves to supply the germ cells [8,16,30,31,32].

To gain insight into the attendant morphological transformations during sexual maturation along the entire body axis, we conducted an analysis of somatic and visceral muscles remodeling in anterior versus posterior-located segmental regions from atokous worms versus epitokous male and female animals of *P. dumerilii*. In both anterior and posterior regions, all longitudinal muscles are significantly reduced after sexual maturation, which is associated with a strong atrophy and dedifferentiation of this type of somatic muscles. The transversal muscles (tm), which are responsible for the constant movements of the epitokous paddle-like parapodia during swarming, show additional signs of remodeling. They swell and are severely hypertrophied, which is mainly limited to segments posterior to the epitokous region, where we have also noticed massive vascularization to supply the muscles, and thus ensure constant swimming during swarming. The tm fibers are ultrastructurally characterized by their strongly multiplicated mitochondria, which are responsible for energy supply during the swarming phase, and they may suggest that apoptosis plays a role as mediators for remodeling and sarcolysis during sexual maturation. All of the somatic muscles examined here are histolyzed to varying degrees, with the final sarcolysis being mediated by eleocytes. As with the longitudinal muscles, the number of visceral muscles is also reduced, suggesting that the general muscle breakdown during sexual maturation is independent of muscle type. 

## 2. Materials and Methods

### 2.1. Animal Material

**Figure 1:**
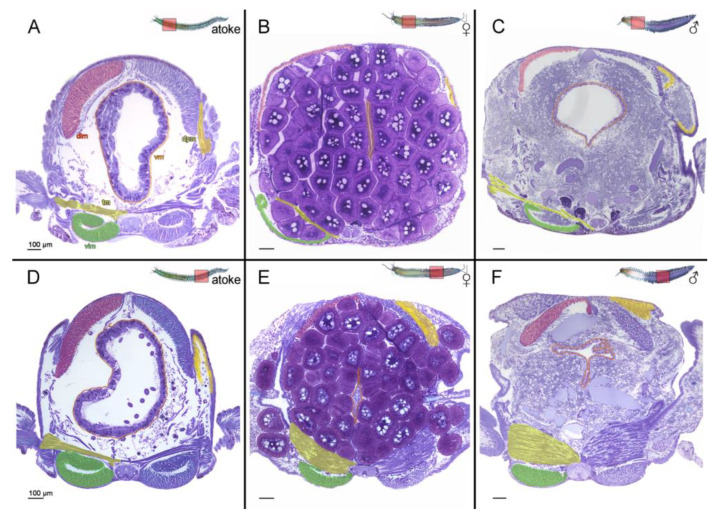
Changes in the morphology of the somatic muscles in the anterior and posterior segments after sexual maturation. (**A**–**F**) Cross-sections of whole *Platynereis* segments displaying all muscles types in immature atokous (**A**,**D**), in female epitokous (**B**,**E**), as well as in male epitokous animals (**C**,**F**). (**A**–**C**) Anterior and (**D**–**F**) posterior-located segments of the same worm. (**A**,**D**) The atokous musculature consists of a thin outer layer of circular muscles (not labeled), the dorsal (dlm red) and ventral longitudinal muscles (vlm green), the transversal muscles (tm yellow), the dorsal parapodial muscles (dpm light yellow), and the visceral musculature surrounding the midgut (vm orange), each marked in one hemisegment. (**B**,**C**,**E**,**F**) Beside the formation of the respective germ cells, the degeneration of the midgut is obvious after the maturation. Longitudinal and transversal muscles are differently remodeled during metamorphosis in dependency to their anterior-posterior position along the body axis. (**A**–**C**) In anterior segments, the dlm and vlm muscles are strongly reduced in thickness and appear much less compact at the end of maturation, while transversal muscles appear unchanged. (**D**–**F**) The dlm and vlm muscles are also reduced in posterior segments, but to a lesser degree. Whereas, in contrast, the transversal muscle complex appears to be very thickened and swollen, and displays an inflated and thus hypertrophied overall morphology. Abbr.: dlm dorsal longitudinal muscles, dpm dorsal parapodial muscles, tm transversal muscles, vlm ventral longitudinal muscles, vm visceral muscles. Scale bars = 100 µm.

**Figure 2:**
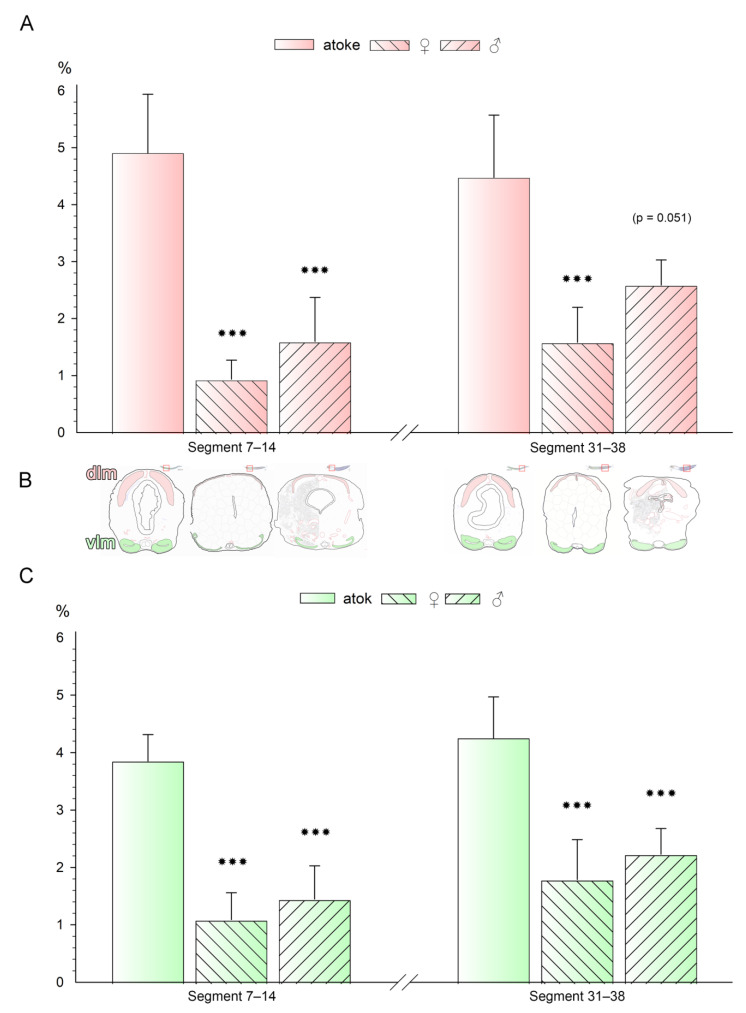
Dorsal and ventral longitudinal muscles are significantly reduced in anterior and posterior segments after maturation. (**A**,**C**) The quantification of the comparative measurements of the total longitudinal muscle size in the (**A**) dorsal or (**C**) ventral longitudinal muscles of atokous, female (♀) and male (♂) individuals. Data were obtained from four replicate measurements of each individual. Total numbers (n) of counted individuals: atokous n = 7, ♀ n = 8, ♂ n = 8 (Supp. Data Sheet). Means and standard deviations of the means are shown. Significance levels were determined by one-way ANOVA for groups within the anterior or posterior segments (n. s. *p* = 0.051, *** *p* < 0.001). (**B**) Cartoons displaying the measurements of dorsal and ventral longitudinal muscles in each section in relation to the total size of each cross-section in atokous, female, and male individuals.

**Figure 3:**
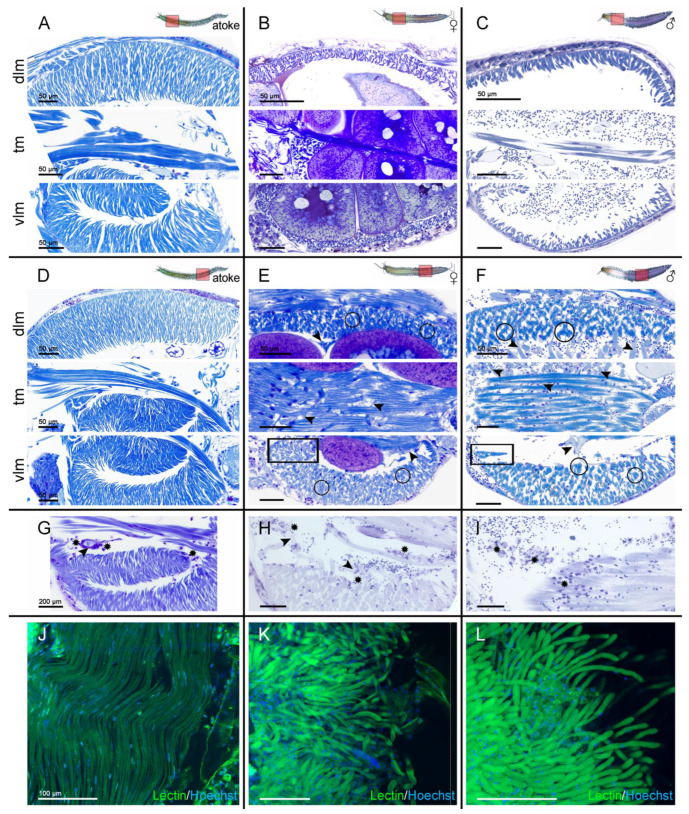
Histological analysis of the remodeling of the different muscle groups. (**A**–**F**) Toluidine blue-stained semi-thin sections of segmental parts derived from cross-sections with magnifications of the main muscles types (**A**,**D**) in atokous, (**B**,**E**) in female, as well as (**C**,**F**) in male epitokous animals in (**A**–**C**) anterior and (**D**–**F**) posterior-located segments of the same worm. (**A**,**D**) The atokous musculature consists of the dorsal (dlm) and ventral longitudinal muscles (vlm), as well as the transversal muscles (tm) shown in one hemisegment. (**B**,**C**,**E**,**F**) Longitudinal and transversal muscles are differently remodeled during maturation in dependency to their anterior-posterior position along the body axis. (**B**,**C**) In the anterior segments, the fibers of the dlm and vlm muscles apparently detach from the basement membrane, drop into the coelom cavity, and are greatly reduced in size, so that their total thickness at the end of the metamorphosis is much smaller in both sexes, while the transversal muscles appear unchanged. (**E**,**F**) The dlm and vlm muscles are also reduced in size and density in posterior-located segments of both sexes, but exhibit additional bundling (circles) of muscle fibers and a massive amount of blood vessels (arrowheads). Only the double layered part of the vlm (rectangles) is remodeled like the anterior-located vlm muscles. In contrast, the transversal muscle complex displays a swollen overall morphology. (**G**–**I**) Small eleocytes (asterisks) invade the longitudinal muscles and the distal areas of the transversal muscles, adhere to the vessels (arrowheads), and appear to move along them towards the coelom cavity, where they enlarge. (**J**–**L**) Segmental parts of ventral longitudinal muscles in atokous (**J**), in female (**K**), as well as in male (**L**) epitokous animals in posterior-located segments. (**J**) Atokous posterior muscles show no signs of blood vessels, (**B**,**C**) while the posterior muscles in epitokous females and males become strongly overgrown by the newly formed vessels and are characterized by WGA Lectin (green) labeling co-stained with Hoechst (blue) to mark the corresponding nuclei. Abbr.: dlm dorsal longitudinal muscle, tm transversal muscle, vlm ventral longitudinal muscle. Scale bars = (**A**–**F**) 50 µm, (**G**–**I**) 200 µm and (**J**–**L**) 100 µm.

**Figure 4:**
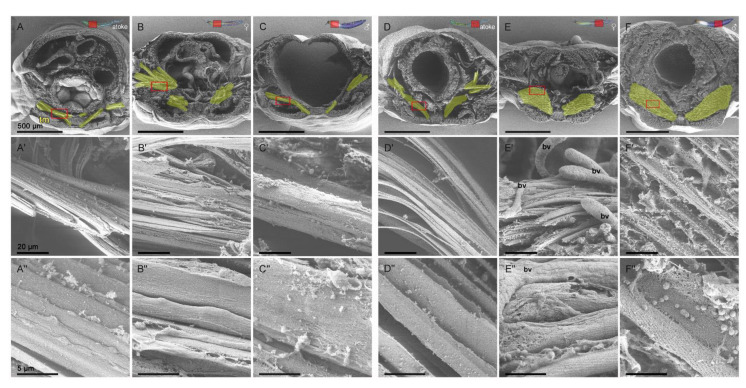
Sexual maturation similarly affects the ultrastructure of transversal muscles along the body axis in scanning electron microscopy (SEM). (**A**–**F**) Overview of the used cross-sections with transversal muscles (tm) marked in yellow and (**A’**–**F”**) ultrastructure of the muscle surface, which were revealed by SEM of anterior and posterior cross-sections of the same worm. (**A**–**C**) While only minor modifications in the complete tm complex were found in the anterior segments, (**D**–**F**) there is an extreme remodeling in the form of strong inflation of the whole muscle complex in the posterior segments. (**A’**,**A”**–**C’**,**C”**) The surface of the anterior tm fibers appear fenestrated and covered with small holes, (**E’**,**E”**,**F’**,**F”**), which is more pronounced in the posterior segments. The tm show signs of cytolysis, i.e., surface dissolution and disintegration, which is also reflected in the release of large quantities of spherical structures covering the transversal fibers. In addition, an overall growth of blood vessels (bv) is detectable. Abbr.: bv blood vessel. Scale bars = (**A**–**F**) 500 µm, (**A’**–**F’**) 20 µm and (**A”**–**F”**) 5 µm.

**Figure 5:**
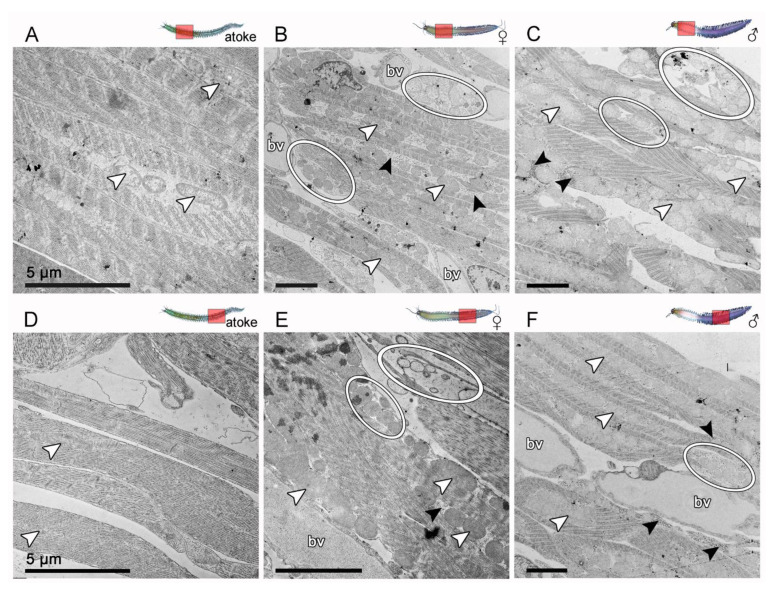
Sexual maturation similarly affects the ultrastructure of transversal muscles along the body axis in transmission electron microscopy (TEM). (**A**–**F**) Ultrastructure of transversal muscles revealed by TEM. Transversal muscles in (**A**,**D**) in atokous, (**B**,**E**) in female epitokous, as well as (**C**,**F**) in male epitokous animals in (**A**–**C**) anterior and (**D**–**F**) posterior-located segments. (**A**,**D**) Atokous tm in the muscle fibers show a clearly pronounced contractile apparatus and only single mitochondria, which are located in the central part of the fibers (white arrowheads). (**B**,**C**) After maturation, the contractile elements of the anterior tm lie on either side of the strongly multiplicated mitochondria (white arrowheads). In some muscles, the contractile apparatus is stronger reduced (circles) and accompanied by an accumulation of glycogen grains (black arrowheads). In addition, an overall growth of blood vessels (bv) is detectable. (**E**,**F**) The ultrastructural changes in the tm fibers of the posterior segments are similar to those of the anterior segments, except for the greater increase in blood vessels (bv). Abbr.: bv vessel. Scale bars = (**A**–**F**) 5 µm.

**Figure 6:**
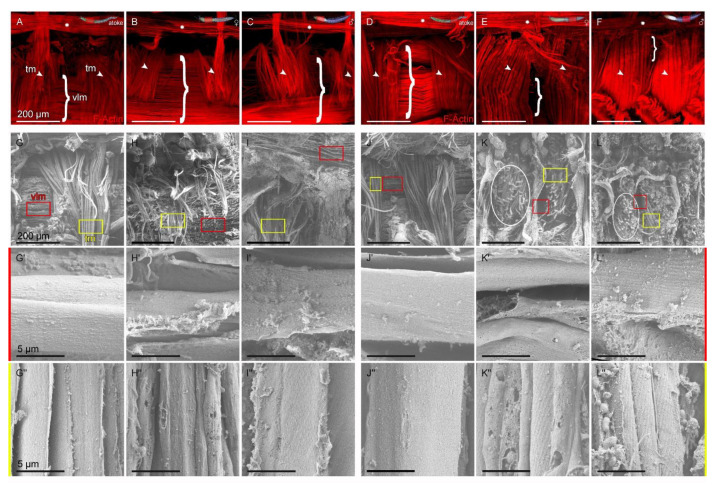
Ultrastructural comparison of ventral longitudinal and transversal muscle remodeling. (**A**–**F**) Overview of Phalloidin-stained ventral muscle filets with ventral longitudinal muscles (vlm, brackets) in parallel to the axochord (asterisks) and perpendicular running transversal muscles (tm, arrowheads) from anterior and posterior segments of the same worm. (**G**–**L**) Low magnification of the ventral muscles with vlm (red boxes) and tm (yellow boxes) in anterior and posterior segments highlighting the massive amount of vessels (circles in **K**,**L**) in the posterior vlm and tm after maturation shown by scanning electron microscopy (SEM). (**G’**–**L’**) High magnification SEM of the vlm display bulges and small holes in anterior (**H’**,**I’**) or small holes and degenerating muscles in posterior segments (**K’**,**L’**) after sexual maturation, while (**G”**–**L”**) the high magnification of the tm reveal strong surface changes (**H”**,**I”**) often combined with resolution of the overall structure (**K”**,**L”**). Abbr.: tm transversal muscles, vlm ventral longitudinal muscles. Scale bars = (**A**–**L**) 200 µm, (**G’**–**L”**) 5 µm.

**Figure 7:**
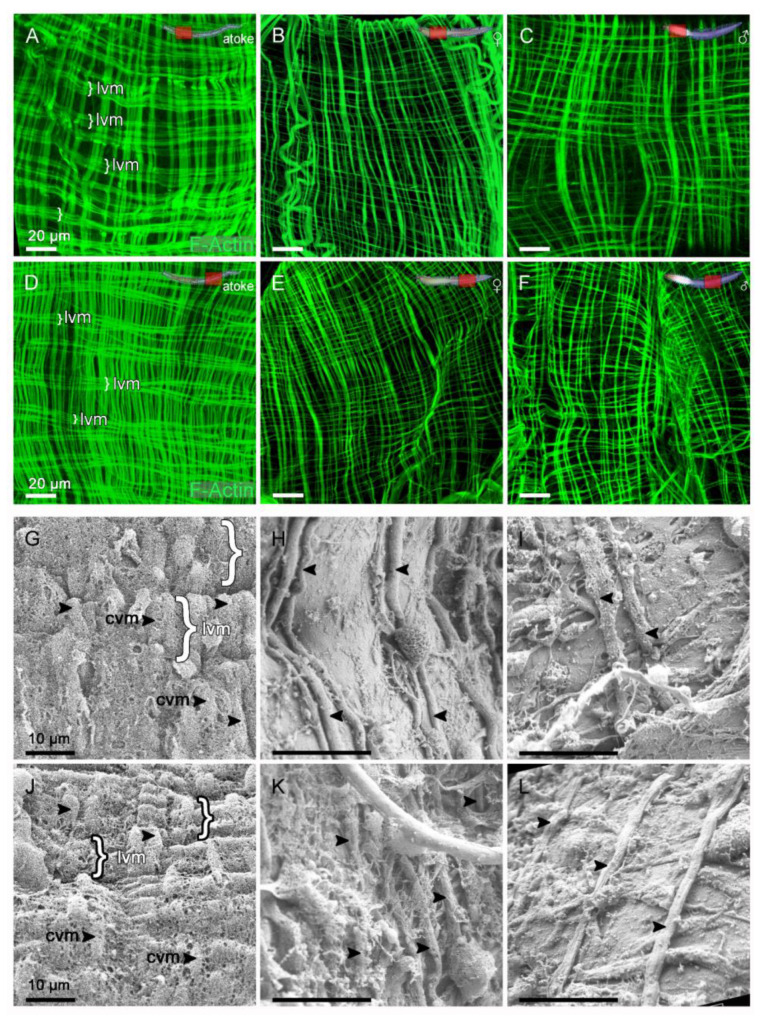
The number of visceral muscles and their coelothelial coverage is greatly reduced after sexual maturity. (**A**–**F**) The general morphology of the visceral muscles in (**A**,**D**) atokous, (**B**,**E**) female, as well as in (**C**,**F**) males in (**A**–**C**) anterior and (**D**–**F**) posterior segments of the same worm revealed by Phalloidin staining after spawning (F-Actin in green). (**A**,**D**) The atokous visceral musculature consists of a thin layer of circular and longitudinal muscles surrounding the midgut, whereby the longitudinal muscles are especially grouped into bundles (lm, brackets). (**B**,**C**,**E**,**F**) After the maturation, this network seems to have loosened up and the longitudinal muscles are more evenly distributed. (**G**–**L**) The ultrastructure of visceral muscles revealed by scanning electron microscopy (SEM). (**G**,**J**) The atokous visceral muscles, (**H**,**K**) female, as well as (**I**,**L**) male epitokous muscles in (**G**–**I**) anterior and (**J**–**L**) posterior segments of the same worm. (**G**,**J**) High magnification SEM of the atokous visceral muscles mainly reveals the parallel aligned circular muscles (cm, arrowheads) and the longitudinal muscle bundles (lm, brackets) both covered with a fenestrated coelothel. (**H**,**I**,**K**,**L**) After maturation, only circular and longitudinal muscles that are further apart can be found under the most severely dissolved coelothel. Abbr.: cvm circular visceral muscle, lvm longitudinal visceral muscle. Scale bars = (**A**–**F**) 20 µm, (**G**–**I**) 10 µm.

*P. dumerilii* were kept using the culture method described by Hauenschild and Fischer [2]. Histological analyses were carried out on atokous animals with at least 60 and at most 75 segments, and were used at these lengths for all experiments. Epitokous females and males were dissected just prior to spawning when they were clearly identifiable, and therefore, fully maturated (Figure 1, Figure 2 and Figure 3 and Figures S1 and S2). The SEM and TEM analyses were also performed on animals of this size and before spawning (Figure 4, Figure 5, Figure 6 and Figure 7, and Figures S3 and S4). However, while we used animals of the same size for Phalloidin staining and Lectin labeling, we fixed these matured individuals immediately after spawning to avoid the bulk of oocytes and sperm in the body cavity (Figure 3J–L, Figure 6A–F and Figure 7A–F, and Figure S3A–F). Whenever possible, all results from anterior and posterior analyses have been presented on images of the same animal.

### 2.2. Preparation and Fixation

The different *Platynereis* specimens (young atokous worms, as well as epitokous females and males) were sedated in 3.75% MgCl_2_ in natural seawater (1:1). For histological sections, samples were dissected in segmental blocks from anterior and posterior parts (Segments 7–14, and Segments 31–38, Figure S1). However, for filet preparations and SEM cross-sections, we often used more than seven segments. For the fixation of histological sections, filets, Phalloidin staining, and Lectin detection 4% paraformaldehyde in phosphate buffered saline (PBS, pH 7.4) was used overnight at 7 °C, followed by 3 × 20 min washing in PBS + 0.1% Tween 20 (PTw). For filet preparations, the segment pieces were dorsally cut after 30 min of fixation time and flat spread, without stretching the tissues, with the help of Minutien needles. This was followed by very gentle rinsing with a pipette to remove the gametes, and fixation was continued overnight. The fixation for scanning electron microscopy (SEM) was 2.5% glutaraldehyde (EM grade) in PBS pH 7.4 for 3 h at room temperature and at least three washing steps for 20 min in PBS, while the primary fixation for transmission electron microscopy (TEM) was in glutaraldehyde (2.5%) in PBS pH 7.4 for 1.5 h at room temperature.

### 2.3. Section and Histology

For an overview of the muscle remodeling process during sexual maturation, we prepared histological sections. For this purpose, the animals were divided into two segmental blocks ranging from segments 7–14 to 31–38. Fixation and washing were followed by dehydration in ethanol series and embedding in Technovit 8100 (Kulzer, Hanau, GER). The sections were always cut from the anterior most front to 5 µm on a rotary microtome (Leitz, Wetzlar, GER) using a D-knife and stained in toluidine blue (0.1% toluidine blue and 0.1% sodium tetraborate in distilled water and filtered before use). Slides were stained in toluidine blue for 2 min, differentiated in distilled water, dehydrated in xylene, dried, and mounted in Entellan (Merck, Darmstadt, GER). Sections were analyzed and documented on an Olympus BX 51 microscope equipped with a Colorview 12 camera and imaging software AnalySIS (Olympus, Shinjuku, Japan). 

### 2.4. Scanning Electron Microscopy (SEM)

Fixed and washed samples were dissected in wax bowls and subsequently dehydrated in ethanol series (50%, 70%, 80%, 90%, and 2 × 100% ethanol for 10 min each). After critical point drying (Balzers CPD 030, Leica, Wetzlar, GER) at 40 °C, the samples were mounted on stubs, sputtered with gold (Balzers SCD 004, Leica, Wetzlar, GER), and studied using electron microscopes (DSM982, Zeiss, Oberkochen, GER and FEI XL30, Philips, Amsterdam, NL). Acceleration voltage was set to 3 kV (Zeiss) and to 15 kV (Philips).

### 2.5. Transmission Electron Microscopy (TEM)

Fixed and 3 × 15 min in PBS washed samples were postfixed in 1% OsO4 (in 0.1 M sodium hydrogen carbonate buffer, pH 7.5) for 1 h at room temperature, washed 2 × 10 min in 0.2 M phosphate buffer, and subsequently dehydrated in ethanol series (50%, 70%, 80%, and 90% for 20 min each, as well as 2 × 100% ethanol for 30 min each). Dehydrated samples were infiltrated with propylene oxide for 3 × 20 min and then overnight. After changing the propylene oxide again for 30 min, the samples were incubated overnight in 1:1 propylene oxide and freshly mixed Araldite, followed by incubation in pure Araldite overnight. All steps were performed at room temperature. After re-incubation for 30 min in fresh Araldite, the samples were embedded and aligned in fresh Araldite for polymerization at 60 °C for 72 h. Ultra-thin sections (60–70 nm) were cut using an ultramicrotome (Leica, Wetzlar, GER) with diamond knife. Sections were stained with 1% aqueous uranyl acetate and lead citrate solutions, mounted on grids, and imaged using an EM 912 AB transmission electron microscope (Zeiss, Oberkochen, GER) equipped with a CCD camera. iTEM software (Olympus, Shinjuku, Japan) was used for image acquisition.

### 2.6. Phalloidin Staining and Lectin Labeling

Fixed *Platynereis* specimens were incubated overnight with Phalloidin or with FITC conjugated WGA Lectin (1:200, Sigma-Aldrich, St. Louis, USA) in PTw at 7 °C and rinsed once, followed by 3 × 10 min washing in PTw at room temperature in the case of Phalloidin detection and counterstained with Hoechst (1:1500 from a 5 mg/mL stock solution, Sigma-Aldrich, St. Louis, USA). Samples were embedded in Fluoromount-G (Southern Biotech, Birmingham, USA) before visualization with a confocal microscope (TCS SP2, Leica, Wetzlar, GER). For visualization of F-Actin in red excitation, we used TRITC-conjugated Phalloidin (1:500 from a 0.2 mg/mL stock solution solved in Methanol, Sigma-Aldrich, St. Louis, USA) and, for green excitation, the Phalloidin-iFluor 488 reagent CytoPainter (1:2000, abcam, Cambridge, UK). 

### 2.7. Muscle Measurements and Statistical Analysis

To determine the extent of muscle remodeling during sexual maturation, we used morphometry of the cross-sections (Figure 1; Figure 2B; Figure S2) to measure the muscle size of the dorsal and ventral longitudinal muscles (Figure 2). The AnalySIS software tool (Soft Imaging System GmbH, Muenster, GER) was used to generate the measurements (Supp. Muscle Measurements) of segmental areas from microscope images, which was carried out for each individual on four cross-sections (replicates) of anterior and posterior segmental blocks. For each cross-section, we determined the area of the dorsal and ventral longitudinal muscles in relation to the total area (Figure 2B). These relationships were compiled in a dataset, which was then statistically evaluated (Supp. Data Sheet). Therefore, the analyzed data set consisted of four measurement replicates for each segmental part with a total number of seven atokous, as well as eight female and eight male animals. Statistics were performed with SigmaPlot 14.0 software (Systat Software Inc.; Chicago, USA). Post hoc analysis for multiple comparisons (one-way ANOVA, Holm-Sidak method with a significance level of *p* = 0.05) was used to analyze the distribution of the three different samples (atokous, female and male animals). 

## 3. Results

### 3.1. Somatic Muscles Are Differently Remodeled in Dependency to Their Position along the Body Axis during Sexual Maturation of Platynereis

In order to gain insight into the underlying transition processes of muscle changes during sexual maturation, we examined the muscle morphology by analyzing histological cross-sections of atokous versus epitokous worms (Figure 1). Due to the formation of the atokous/epitokous border between segments 15–22 [5], we generated the anterior sections between the 7th–14th segments (S 7–14) and the posterior sections between the 31st–38th (S 31–38) segments (Figure S1). 

Atokous segments are characterized by the same muscle composition and morphology in anterior as well as posterior segments (Figure 1A,D), which consist of a thin layer of circular muscles located directly below the epidermis (not labeled), whereas the much stronger longitudinal muscles are divided into dorsal (dlm red) and ventral groups (vlm green). The transversal muscles (tm yellow), which extend from attachment sides dorsal and ventral of the axochord to the parapodia, as well as the dorsal parapodial muscles (dpm light yellow) are responsible for parapodial locomotion, while the visceral muscles (vm orange) consist of both longitudinal and circular fibers and envelope the midgut. 

During sexual maturation, the formation of the respective germ cells and the degeneration of the midgut [2] are obvious in all anterior and posterior segments of both sexes (Figure 1B,C,E,F). Both dorsal and ventral longitudinal muscles are strongly reduced in their overall thickness in the anterior segments of both sexes, and appear to be much less compact at the end of maturation (Figure 1B,C; Figure 2). These muscles are also reduced in their thickness in the posterior segments, but to a lower degree (Figure 1E,F; Figure 2). All longitudinal muscles are severely atrophied and reduced in density of fiber numbers after maturation.

The transversal muscles (tm), as well as the dorsal parapodial muscles, differently develop during maturation. While they maintain roughly the same thickness in anterior segments and thus appear unchanged (Figure 1B,C), the whole muscle complexes are strongly thickened, swollen, and distended, and thus hypertrophied in the posterior segments (Figure 1E,F), which shows that longitudinal and transversal muscles are differently transformed during sexual maturation. The different degrees of muscle breakdown and modification appear to depend on segmental affiliation, and therefore reflect the division of tasks of the various body regions after maturation. 

These results indicate alternative developmental programs for atokous and epitokous regions with dedifferentiation and atrophy for the longitudinal muscles (dlm and vlm) on the one hand, and hypertrophy and the possibly some kind of redifferentiation for both the transversal muscles and the dorsal parapodial muscles on the other. 

### 3.2. Dorsal and Ventral Longitudinal Muscles Are Significantly Reduced in Anterior and Posterior Segments after Maturation

To determine the extent of the muscle remodeling during maturation, we used morphometry of the cross-sections (Figure 1; Figure 2B; Figure S2) to measure the average muscle area in anterior and posterior segments by determining the proportion of muscle on the total segment area (Figure 2, Supp. Muscle Measurements, Supp. Data Sheet).

The average value for the dorsal longitudinal muscles (dlm) accounts for about 4.91%, respectively, 4.48% of the total segment area in anterior versus posterior parts of atokous animals, while this proportion drops after maturation to 0.94% in females, respectively, 1.60% in males in anterior segments and to 1.58%, respectively, 2.59% in posterior segments. Whereby the reduction in the front segments is greater than that in the rear segments, and is also different depending on gender; in females, the remodeling of the dlm in anterior and posterior segments is slightly more pronounced than in males (Figure 2A). 

The average value for the ventral longitudinal musculature (vlm) takes up a smaller proportion of about 3.85%, respectively, 4.25% of the total segment area in atokous animals compared to the dlm, while this proportion decreases to about 1.09%, respectively, 1.45% in front segments, and to 1.79%, respectively, 2.23% in rear segments after maturation. Again, depending on the position along the anterior-posterior axis, this is slightly more pronounced in the anterior segments than in the posterior segments (Figure 2C). Here, only a slightly stronger remodeling in females compared to males is noticeable. 

Overall, the longitudinal muscles decrease in anterior as well as in posterior segments of *Platynereis dumerilii*, which is slightly more pronounced in female animals (Figure 2A,C).

### 3.3. Histological Analysis of the Remodeling of the Different Muscle Groups

In order to gain insight into the observed differences in muscle remodeling during maturation, we analyzed the muscle groups in more detail (Figure 3) using the histological cross-sections. The dorsal and ventral longitudinal muscles (dlm and vlm) of atokous animals consist of parallel lamellar fibers, the outer edges of which are attached to a basement membrane (BM) and the inner edges of which border the coelom, whereby the ventral BMs protrudes from the body wall into the body cavity, and thereby, the ventral longitudinal muscles are built up in a double layer, which is achieved by the lengthening and folding of these ventral BMs. The transversal muscles (tm) run above the vlm and are stretched between BMs on the axochord and parapodia (Figure 1A,D; Figure 3A,D). 

In the anterior segments of both females and males, the breakdown of the ventral and dorsal longitudinal muscles proceeds in the same way, i.e., the muscle fibers both decrease in size and partially detach from the basement membrane (BM), which, in addition to size, also greatly decreases their overall density (Figure 3B,C). As a result, many spindle-shaped sarcolytes are found in the coelom as remnants of muscle breakdown (Figure 3G–I). The transversal muscle complex appears unchanged in the anterior segments of both sexes (Figure 1B,C; Figure 3B,C). 

In the posterior segments of both sexes, the breakdown of dorsal and ventral longitudinal muscles takes place in a slightly different way, i.e., the muscle fibers also decrease in size and density, but, at the same time, bundles of several closely connected fibers are formed (circles in Figure 3E,F). These fiber bundles consist of 3–10 severely shrunken but closely interconnected fibers, with many surrounding gaps of former muscle fibers, through which now small blood vessels grow (arrowheads in Figure 3E,F). Interestingly, as in the anteriorly located longitudinal muscles, the degradation of the adjacent, folded, double-layered inner portion of the vlm (rectangles in Figure 3E,F) exclusively occurs through shrinkage, atrophy, and detachment.

The transversal muscles (tm) of the posterior segments of both sexes show the greatest changes after the maturation of all muscle groups examined here. Many fibers swell, resulting in remaining hypertrophied fibers, from which small, blind-ending blood vessels protrude (arrowheads in Figure 3E,F), resulting in a severely loosened overall structure. In order to visualize the strong vascularization of posterior muscles, we use fluorescein-labeled Lectin (wheat germ agglutinin, WGA) to label the newly formed vessels. Labeled blood vessels are not detectable in the ventral longitudinal muscles of the posterior segments of atokous animals (Figure 3J), while Lectin-positive vessels are particularly prominent in the posterior segments (Figure 3K,L), and also in the anterior segments of epitokous animals (not shown). They are growing with blind endings through the dlm, vlm, and tm (arrowheads in Figure 3E,F), and are formed further away from the body wall in the coelomic cavity and appear most pronounced in males (Figure 3F,L). 

We also detected, in our histological sections, several small, dark toluidine blue-stained, single cells in the musculature of anterior and posterior segments, which we identified as small eleocytes and followed their differentiation during muscle histolysis in posterior segments (Figure 3G–I). Small eleocytes invade the longitudinal muscles and the distal areas of the transversal muscles. They closely adhere to the vessels, seem to move along them towards the coelom cavity, and increase strongly in size (asterisks in Figure 3G–I). In the coelom, they are found in large quantities, close to the sarcolytes of the longitudinal muscles. Although the number of eleocytes in the anterior segments appears somewhat lower, their final sarcolysis is apparently also mediated by eleocytes.

In summary, transversal muscles in anterior segments do not appear remodeled after maturation, but the anterior longitudinal muscles are severely reduced in size and number, while in the posterior segments, secondary bundling of longitudinal fibers, as well as the thickening of transversal muscles, can be observed along with strong degradation processes in longitudinal muscles, and also partially in transversal muscles. It appears that the muscles of the anterior segments mainly dedifferentiate, with the final sarcolysis mediated by eleocytes, while the muscles of the posterior segments show additional signs of redifferentiation, possibly caused by the strong posterior vascularization.

### 3.4. The Ultrastructure of Transversal Muscles along the Body Axis Is Similarly Affected by Sexual Maturation

Due to the different appearance of the posterior transversal muscles (tm) after maturation compared to the longitudinal muscles (Figure 1, Figure 3) and the difficulties in measuring their muscle size due to their complex spatial expansion, we looked for a different approach in order to obtain further insights into the structural morphology of transversal muscle remodeling, and therefore, we carried out ultrastructure analysis with scanning electron microscopy (SEM) on cross-sections (Figure 4), as well as transmission electron microscopy (TEM) on sections from segmental pieces (Figure 5), which we both prepared at the end of the maturation process after final gender recognition and before spawning. 

While the size of the transversal muscles (tm in yellow) in anterior segments (Figure 4A–C) seems to remain almost constant after maturation and mainly depends on the position of the section in relation to the parapodium, the surface of the individual muscle fibers in anterior epitokous animals appears strongly fenestrated and covered with more small holes (Figure 4B’,B”,C’,C”; Figure 6H”,I”) compared to the mostly regular surface of the anterior atokous tm (Figure 4A’,A”; Figure 6G”). This difference increases into a serious change in the tm in posterior segments in both sexes after maturation (Figure 1E,F; Figure 3E,F; Figure 4E,E’,E”,F,F’,F”; Figure 6K,K’,L,L’). The entire transversal muscle complex appears severely thickened and bloated; i.e., severely structurally altered. Despite the thickening, many muscles appear to be damaged in the process of maturation (Figure 4E”,F”; Figure 6K’,K”,L’,L”), and the many newly formed blood vessels in between are clearly visible (bv and circles in Figure 6K,L). Sometimes, the entire surface of the muscles seems to be dissolved and is then often ultrastructurally covered with small spherical structures (Figure 4E”,F”; Figure 6K”,L”). These structures were not found in atokous animals in the area of cut edges (Figure 4A–A”), which indicates the absence of these structures or a changed surface structure of atokous muscles. However, since they are common in adult animals, we wanted to analyze their structure and if they could be released during the regular maturation process.

To examine transversal muscle (tm) remodeling in detail, we performed transmission electron microscopy (TEM) analyses of anterior (Figure 5A–C) and posterior (Figure 5D–F) atokous versus epitokous animals. Atokous tm consist of parallel fibers that extend between the basement membranes (BMs) of the axochord and the parapodia, and show a clearly pronounced contractile apparatus and only a few isolated mitochondria, which are located in the middle of the fibers (white arrowheads in Figure 5A,D). After maturation, the transversal fibers appear to be greatly expanded in both males and females by large amounts of mitochondria occupying a central position between the contractile elements on either side of the muscle fibers. Some fibers, often located near newly grown blood vessels (bv), have a completely reduced contractile apparatus and are additionally filled with glycogen grains (black arrowheads) and appear to disintegrate their plasma membranes (circles in Figure 5B,C,E,F). This makes them look like doomed muscles, the remnants of which are found later in the coelomic cavity as sarcolytes. The overall structure of tm seems to have loosened up after maturation, and to be interspersed with newly formed vessels (Figure 5B,C,E,F). Interestingly, in posterior segments of both females and males, the remodeling of the tm fibers seems to proceed in the same way as in the anterior segments, with numerous mitochondria lying in the midst of the contractile elements (Figure 5E,F). All accompanying ultrastructural changes of the posterior fibers, such as the dissolution of some fibers, appear similar to those of the anterior segments, except for the stronger increase in blood vessels in the posterior segments. This points to the differing role of the tm in posterior segments for the continuous parapodial movements during reproductive swarming behavior. Although the posterior tm show the greatest changes in our histological analyses, they do not show ultrastructural differences in their general remodeling compared to the anterior tm. 

In conclusion, despite the histological differences observed between anterior and posterior transversal muscles, we found that these muscles undergo similar ultrastructural remodeling during maturation by multiplying the number of their mitochondria. Thereby, the muscle cells increase in volume, while in some muscles, the contractile apparatus completely disappears and the fibers are eventually filled with glycogen grains and mitochondria before they are cytolized (circles), possibly to create space for the newly emerging vessels. 

As we cannot confirm, with the TEM analyses, the high amount of spherical structures observed with SEM, we conclude that this must have something to do with muscle surface changes during the maturation process, resulting in an increased release of membrane vesicles or mitochondria during the SEM preparation steps.

### 3.5. Ultrastructural Comparison of Ventral Longitudinal and Transversal Muscle Remodeling

In order to avoid abnormalities in muscle morphology and ultrastructure due to the sectioning process, we took advantage of filet preparations (Figure 6), which were prepared for scanning electron microscopy (SEM) at the end of the maturation process after final gender recognition, and therefore, before spawning (Figure 6G–L), while the filets for Phalloidin staining were immediately prepared after spawning to reveal the full muscle morphology without large amounts of oocytes and sperm (Figure 6A–F).

The general organization of the ventral musculature (vlm, brackets) running in parallel on both sides (only one side is shown) of the axochord (asterisks), together with the transversal muscles (tm, arrowheads) running perpendicular to it, remains nearly unaffected during the maturation of the anterior segments (Figure 6A–C,G–I). In detail, however, the surface of the maturated anterior vlm seems to have shrunk and often displays bulges (Figure 6H’,I’) compared to the atokous muscle (Figure 6G’), which indicates a reduction in volume of the anterior vlm during the maturation process, and thus, confirms the previous observations and measurements (Figure 1, Figure 2 and Figure 3). The vlm also often show a loose structure with many detached muscles and perforated surfaces, as well as many spherical structures at the edge of the remaining muscles (Figure 6H’,I’), indicating extensive surface alteration during the maturation of vlm fibers. The changes in the anterior transversal muscles (tm) are also characterized by dissolving surfaces, an accompanying relaxation, as well as the already observed surface changes with an often perforated surface (Figure 6H”,I”) compared to the atokous tm (Figure 6G”). In addition, blind-ending blood vessels can be found in both the vlm and tm of the anterior segments, growing through both muscle types. 

In contrast to the anterior segments, the general organization of the ventral muscles is significantly changed after the maturation process of the posterior segments (Figure 6D–F,J–L), because the vlm are no longer visible due to the overgrowth of the tm, but can still be found below (Figure 1E,F; Figure 3E,F; Figure 6K,L). The fenestrated surface (Figure 6K’,L’) and the presence of many small blood vessels (circles in Figure 6K,L) observed in the ultrastructure of the vlm confirm that the muscle structure appears to be severely compromised after the metamorphosis of the posterior segments (Figure 6K’,L’). The tm also appears to be partially dissolved and some spherical structures are also visible (Figure 6K”,L”). The already observed strong remodeling of the tm with some cytolized fibers after maturation of the posterior segments (Figure 4E’,F’) is also visible here in the dorsal view, and is accompanied by a large number of small blood vessels (bv in Figure 4E’,E” and circles in Figure 6K,L). However, as already mentioned above, it cannot be ruled out that the modified muscle surface after maturation is more sensitive to the preparatory steps for the production of the SEM preparations, in particular the critical point drying, and that this leads to severe disturbances in the muscle ultrastructure of well-matured animals.

In summary, direct comparison only shows gradual ultrastructural differences in the extent of remodeling between vlm and tm, which are likely to mainly depend on the position along the body axis and the degree of sexual maturity of individual animals. 

### 3.6. The Number of Visceral Muscles and Their Coverage with Coelothel Is Reduced after Sexual Maturation

The histolysis of the midgut causes its structural collapse, and thus also presumably the breakdown of the framework for the surrounding visceral muscles. This should be exacerbated by the massive oocyte growth in the female segments and indicates further remodeling of the visceral muscles during the maturation process. These strong morphological changes greatly reduce the size of the entire midgut (Figure S3A–L). However, the remaining visceral muscle network still appears very regular in Phalloidin staining’s performed on dissected midguts after spawning (Figure S3B,C,E,F; Figure 7B,C,E,F). Interestingly, the remaining muscles exhibit a somehow loosened morphology compared to the atokous situation (Figure S3A,D; Figure 7A,D). Especially, the longitudinal muscles no longer show their characteristic bundling (brackets in Figure 7A,D), which indicates further degradation of the enveloping coelothel as well.

Therefore, we also analyzed the visceral musculature in more detail (Figure 7; Figure S3; Figure S4), which has been described as a layer of smooth longitudinal and circular muscle fibers surrounding the midgut [28]. Transmission electron microscopy (TEM) of cross sections additionally shows the covering of the visceral muscles with coelothelial cells (Figure S4). In order to avoid abnormalities in muscle morphology due to the Phalloidin staining’s prepared after spawning, we again used the filet preparations, which were prepared for scanning electron microscopy (SEM) at the end of the maturation process, after final gender recognition, and therefore, before spawning. We found that the longitudinal and circular fibers are collectively covered by a coelothel, which often shows small holes and fissures (Figure 7G,J; Figure S4). The ultrastructure of atokous visceral muscles mainly shows the underlying parallel running circular muscles (arrowheads in Figure 7G,J) and the longitudinal muscles (brackets in Figure 7G,J; Figure S3M,P). After maturation, the number of circular muscles (arrowheads) decreases, and thus, their spacing increases, while longitudinal muscles can only be sporadically identified, which is similar in both the anterior and posterior segments. Interestingly, the coelothel cover is also strongly affected (Figure 7H,I,K,L; Figure S3N,O), which often leads to an almost complete loss in anterior and posterior segments (Figure 7H,I,K,L; Figure S3Q,R). Thus, in addition to visceral muscle reduction, we also show the breakdown of the splanchnic coelothelium during sexual maturation.

## 4. Discussion

### 4.1. Remodeling of Muscle Groups along the Body Axis during Sexual Maturation in Platynereis dumerilii

We have shown that all dorsal and ventral longitudinal muscles in anterior- and posterior-located segments are greatly reduced in size and density during sexual maturation, which is most pronounced in the anterior segments of males, and even more in females. Histolysis of longitudinal muscles generally occurs in many polychaetes, which results in an enlargement of the coelomic cavity [8,9,16,30]. In male *Alitta virens* (formerly in the genus *Nereis*), histolysis reduces all posterior-located longitudinal muscles, but eventually even reaches the anterior segments, while in female worms, anterior muscles remain normal, since the gametes are stored in more posterior regions, where the longitudinal muscles become as modified as much as in males. The greater extent of epitokous modifications in males is associated with the restriction of swarming behavior in *A. virens* to males [9]. Therefore, a changed muscle breakdown must be postulated for *Platynereis*, since both males and females swarm and, accordingly, the posterior-located muscles must be remodeled in both sexes. This general remodeling of the posterior longitudinal and transversal muscles has already been described for *P. dumerilii* as one of the muscular prerequisites for swarming behavior [2,3,6,27]. Since, in *Platynereis* germ cells are also anteriorly found, and these anterior body regions are not needed for burrowing due to their living in tubes, anterior longitudinal muscles should also be histolyzed in order to enlarge the body cavity for the storage of germ cells. The presented results show this severe anterior muscle reduction in both sexes, where it is more pronounced in females, likely due to the greater coelomic enlargement for oocyte storage. In addition, we show that the anterior reduction of the longitudinal muscles is actually greater than the posterior reduction, possibly caused by the excessive remodeling of the transversal muscles in posterior body regions, which require a greater antagonistic musculature in the form of better-preserved longitudinal muscles for their long-term mobility, which is needed during the swarming phase. In this context, the secondary fiber bundles in the degraded posterior longitudinal muscles, together with the newly grown vessels, could be of particular interest, as they could provide the necessary support for the transversal muscles through a secondary consolidation of the strongly atrophied longitudinal muscles. Similar structures can be observed in histological cross-sections of posterior epitokous longitudinal muscles of *Neanthes nubila* (formerly known as *Nereis irrorata*) (Figure 1D in [33]), and therefore, probably fulfill similar functions.

It has been reported by several authors that histolysis in the longitudinal muscles of many polychaetes is generally initiated by invading eleocytes. This separates the muscle lamellae from their connective tissue, indicating a role for the invading eleocytes in extracellular matrix (ECM) degradation, and isolated muscle fibers fall into the coelom, where they are eventually phagocytosed by eleocytes [2,8,16,27,31,32,34]. In male *A. virens*, the remaining fibers become atrophied [9], which seemingly also applies to most of the other analyzed species. The results of our histological analysis confirm this general course. However, the presented results do not allow a decision as to whether invading eleocytes are the cause or the result of muscle breakdown. It cannot be ruled out that there is a general detachment of the muscle fibers from their ECM, triggered by an as yet unknown source, which was supported by Dhainaut [35], who reported that eleocytes from *Alitta* phagocytize only sarcolytes and do not histolyze intact muscle tissue.

Wissocq [33] found, in ultrastructural analysis of posterior segments in *Neanthes nubila*, that epitokous longitudinal muscles are transdifferentiated from atokous fibers. The first sign of longitudinal muscle remodeling is the dedifferentiation of the coelom edge of the lamellar muscle fibers, which is associated with the disappearance of the contractile apparatus and the formation of glycogen particles. As a result, the coelomic edge of the fibers enlarges and in the axial fiber region numerous mitochondria appear next to the glycogen particles, which ultimately leads to the formation of the epitokous longitudinal fiber, which shows a division into a myoplasmic cortex with the contractile apparatus and a sarcoplasmic medulla, which is characterized by multiple mitochondria and glycogen. During our ultrastructural analyses, we noted complete dissolution of some longitudinal and transversal muscle fibers in *Platynereis*. While the epitokous longitudinal muscles in *N. nubila* have a high density, appear highly organized, and display secondary fiber bundles, we found in contrast in *Platynereis* a greatly reduced density and size together with the secondary bundling of the dedifferentiated epitokous longitudinal muscles. Posterior vascularization has also been reported for *N. nubila* [33], which presumably leads to the observed secondary bundling of the remaining longitudinal fibers in posterior segments in both species.

As the posterior parapodia are reorganized into paddle-like body appendages during sexual maturation, a profound reshaping of the involved longitudinal and transversal muscle groups is necessary, for which the transversal muscles must be reorganized into muscles capable of continuous swimming and must be therefore well supplied with blood vessels. Although this redifferentiation of the transversal muscles is considered to be another common feature of muscle remodeling during Heteronereis formation in *P. dumerilii*, the process has been little studied so far and transdifferentiation of the existing muscles has been assumed [1,2]. We have shown that although transversal muscles in the anterior and posterior segments are ultrastructurally similarly transformed during maturation, only the posterior segments display the strongly swollen structure of the transversal muscle complex. Given the same general degradation process, we suspect that the tremendous proliferation of the small blood vessels in the posterior segments that grow through all of the muscles may lead to the amazing swelling of all of the posterior transversal muscles. 

As a result of sexual maturation, *A. virens* changes both the body wall and the digestive tract. While the female gut musculature remains much as in atokous animals, in the male the intestinal mucosa degenerates and the visceral longitudinal and circular muscles of the intestine become much reduced [9]. Also, in *Platynereis*, the midgut is surrounded by smooth muscles [2,28]. Although the midgut is severely atrophied during the maturation process, we show here that the visceral muscles survive with a reduced number, which is more obvious in the posterior-located segments and therefore similar to *Alitta*. Consequently, in *Platynereis*, both striated somatic and smooth visceral muscles are reshaped during the formation of the Heteronereis, which demonstrates the independence of the general remodeling from the underlying muscle type. 

### 4.2. Muscle Histolysis during Sexual Maturation

The sexual maturation and muscle histolysis to supply the growing oocytes with vitellogenin via the sarcolytes-degrading eleocytes in *Platynereis* is induced by the decrease in the recently discovered methylfarnesoate as an essential brain hormone [1,2,4,6,32,36,37,38,39]. Interestingly, the histolysis of larval muscles of *Manduca* is also triggered by changes in hormone titer and results in a large reduction in the size of degenerating fibers in cross-sections. As the fibers shrink, the basement membrane (BM) is wrinkled and phagocytic hemocytes accumulate on the surface and appear to specifically remove the BM [40,41]. Although there is no BM around the muscle fibers described for *Platynereis* [2], we frequently observed wrinkles on the muscle surface in our scanning electron microscopy (SEM) analyses, which are probably caused by the general large size reduction. Furthermore, we found a strong surface alteration of all longitudinal and transversal muscles after maturation, which may lead to an increased surface dissolution and to the release of spherical structures after SEM treatment.

Atokous muscle fibers in polychaetes are characterized by a small number of mitochondria that enormously multiply during epitokous metamorphosis and arrange themselves in the middle of the muscle fibers, thus separating the two contractile flanks of the fibers [18]. In *Platynereis*, we found that all transversal muscles are ultrastructurally remodeled in the same way, in that the mitochondria begin to multiply, the muscle cells increase in volume, and the muscles eventually appear filled with mitochondria. In some muscle fibers, however, the contractile apparatus in the resulting muscle halves is completely degraded and glycogen grains become visible when dissolution of the plasma membranes eventually occurs, possibly triggered and aggravated by the added pressure of the invading blood vessels.

## 5. Perspectives

The extraordinarily high concentration of mitochondria in the epitokous parapodial muscles of various polychaetes has been regarded as a prominent characteristic feature of their transdifferentiation associated with the metabolic performance for continuous swimming movements during germ cell release [2,8,16,18,27,33,42,43,44,45,46]. However, it remains questionable as to why all of the muscles broken down then show the same high amounts of mitochondria, since the anterior muscles in particular no longer seem to play a significant role after the maturation process. Since mitochondria are known to play key roles in activating apoptosis in mammalian cells, as well as in invertebrates [47], we propose that the extraordinary amounts of mitochondria could additionally provide a signal for triggering programed cell death. Furthermore, the unusual massive proliferation of posterior blood vessels could indicate apoptosis-induced vascular remodeling [48].

## Data Availability

All data generated or analyzed during this study are included in the manuscript.

## Supplementary Materials

The following supporting information can be downloaded at: https://www.mdpi.com/article/10.3390/biology12020254/s1, Figure S1: Illustrated origin of the experimental cross-sections from both sides of the atokous/epitokous (a/e) border, Figure S2: Cross-section derived schemes illustrating the muscle thickness of dorsal and ventral longitudinal muscles in anterior and posterior segments in relation to the size of the whole segment before and after maturation, Figure S3: Sexual maturation affects the structure of the midgut and the morphology of the surrounding visceral muscles, Figure S4: Structure and ultrastructure of *Platynereis* visceral muscles, Supp. Data Sheet and Supp. Muscle Measurements.

## Abbreviations

BM basement membrane, bv blood vessels, cvm circular visceral muscles, dlm dorsal longitudinal muscles, dpm dorsal parapodial muscles, ECM extracellular matrix, gc germ cells, lvm longitudinal visceral muscles, mg midgut, SEM scanning electron microscopy, sm suspension muscles, TEM transmission electron microscopy, tm transversal muscles, vlm ventral longitudinal muscles, vm visceral muscles, vnc ventral nerve cord.
