# Supplementary material for: Remodeling of the Platynereis Musculature during Sexual Maturation
Source: Biology (Basel). 2023 Feb 6;12(2):254. doi: 10.3390/biology12020254 (PMC9953025; doi:10.3390/biology12020254)
Supplement: Supplementary file 1 [file biology-12-00254-s001.zip › 5 biology-2147099 Supp. Figures and Supp. Figure Legends.pdf]

Remodeling of the *Platynereis* musculature during sexual maturation

Ina Dahlitz (ORCID: 0000-0002-3414-6118), Adriaan Dorresteijn (ORCID: 0000-0003-2314-1634) and Anne Holz[#] (ORCID: 0000-0002-7028-6548)

Justus-Liebig-Universitaet Giessen, Fachbereich 08 Biologie und Chemie, Institut fuer Allgemeine Zoologie und Entwicklungsbiologie, Heinrich-Buff-Ring 38, 35392 Giessen, Germany.

# Correspondence should be send to: anne.holz@allzool.bio.uni-giessen.de

Supplementary Figures and Supplementary Figure Legends

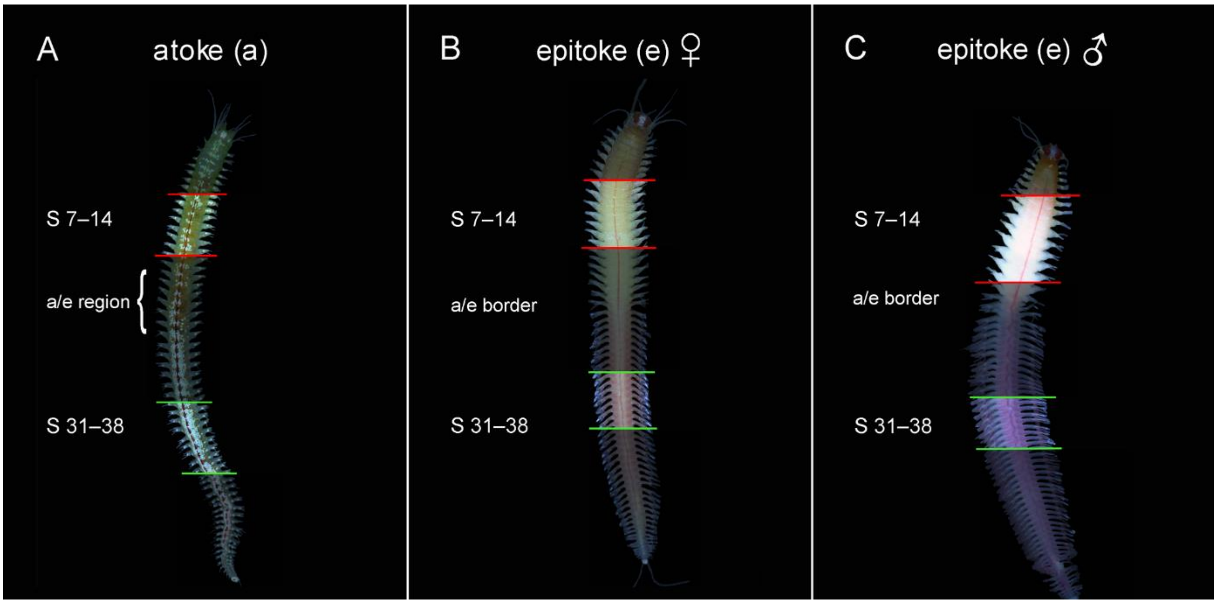

Figure S1: Illustrated origin of the experimental cross-sections from both sides of the atokous/epitokous (a/e) border. (A–C) Anterior cross-sections are from segmental blocks 7–14 (S 7–14), while the posterior cross-sections are from segmental blocks 31–38 (S 31–38) and stemming therefore several segments away from the a/e transition. All cross-sections from the anterior or posterior areas were made from the anteriormost front of the respective regions.

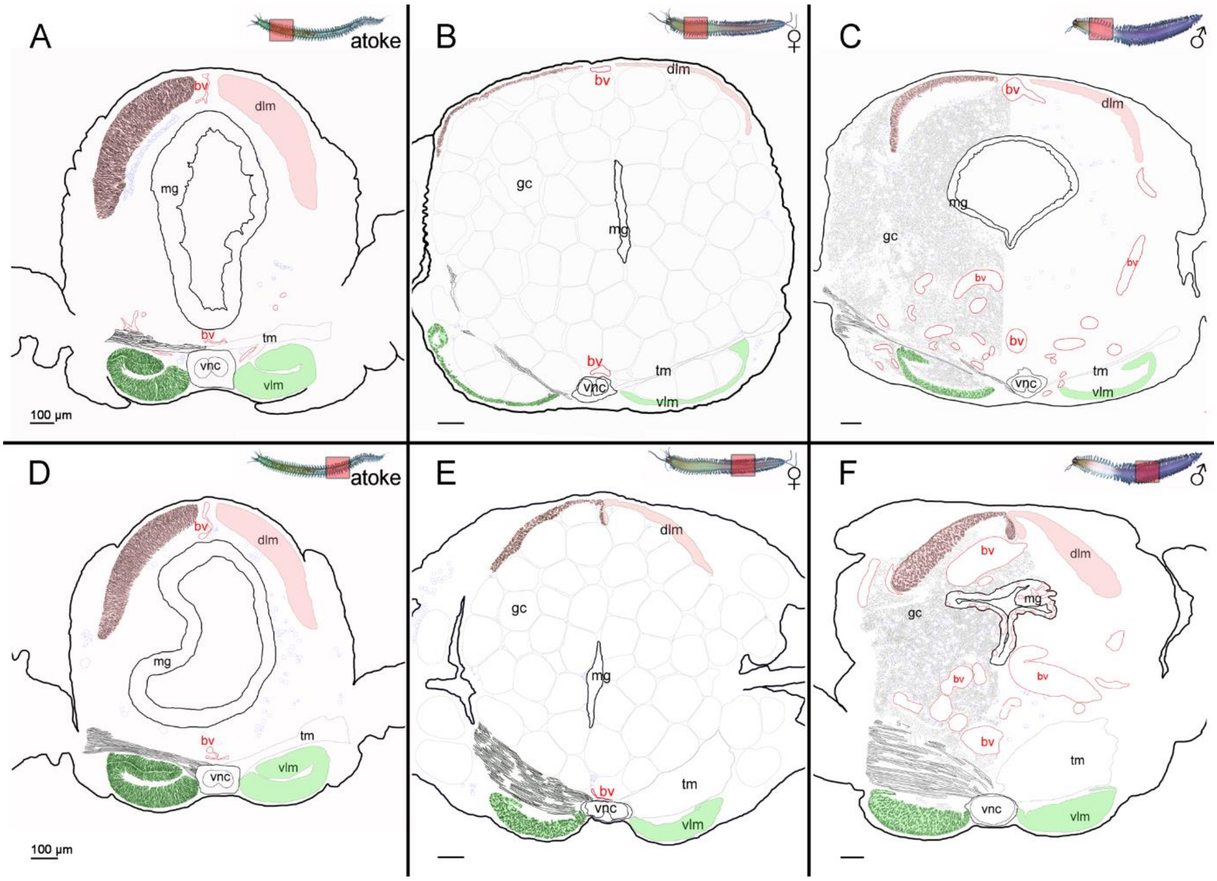

Figure S2: Cross-section derived schemes illustrating the muscle thickness of dorsal and ventral longitudinal muscles in anterior and posterior segments in relation to the size of the whole segment before and after maturation. (A, D) Anterior and posterior atokous segments before maturation. **(B, E)** Epitokous female segments are characterized by a huge amount of oocytes in the body cavity, **(C, F)** while all male epitokous segments are full of sperm. Abbr.: bv blood vessels in red, dlm dorsal longitudinal muscles in pink, gc germ cells, mg midgut, tm transversal muscles, vlm ventral longitudinal muscles in green, vnc ventral nerve cord. Scale bars: 100 µm.

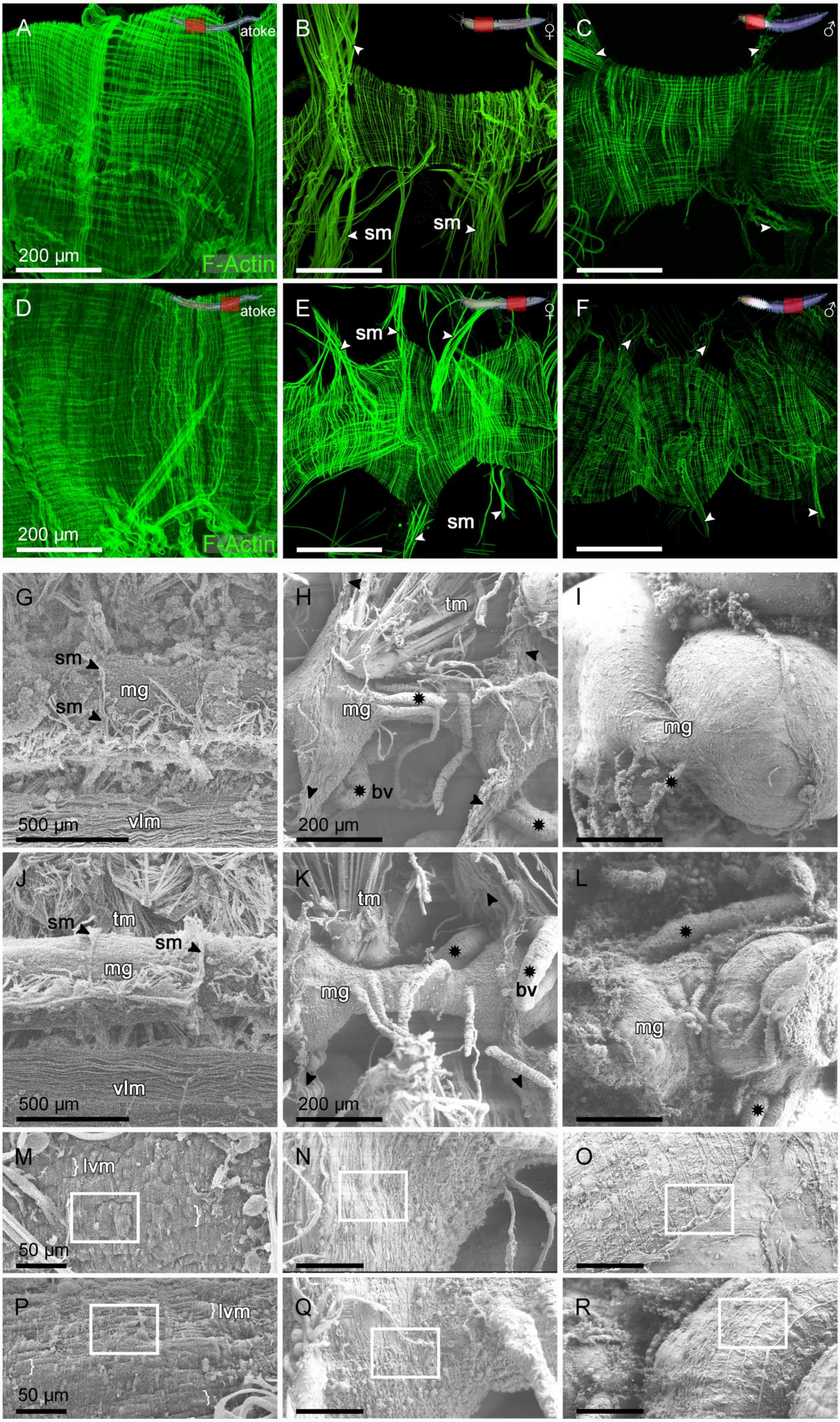
A
atoke
200 µm
F-Actin
B
♀
sm
sm
C
♂
D
atoke
200 µm
F-Actin
E
♀
sm
sm
F
♂
G
sm
mg
sm
500 µm
vlm
H
tm
mg
bv
200 µm
I
mg
J
sm
tm
sm
mg
500 µm
vlm
K
tm
mg
bv
200 µm
L
mg
M
lvm
50 µm
N
O
P
lvm
50 µm
Q
R

Figure S3: Sexual maturation affects the structure of the midgut and the morphology of the surrounding visceral muscles. (A–F) Morphology of F-Actin stained visceral muscles. **(A, D)** Visceral muscles in atokous animals, **(B, E)** female as well as **(C, F)** male epitokous visceral muscles in **(A–C)** anterior and **(D–F)** posterior segments of the same worm. **(A, D)** Visceral muscles consist of inner longitudinal and outer circular muscle fibers, **(B, C, E, F)** which appear loosened up after the sexual metamorphosis, while the midgut itself is greatly reduced in size after the maturation and is connected to the body wall via the suspension muscles (sm) of the dissepiments (arrowheads). **(G–L)** Low magnification scanning electron microscopy (SEM) shows the reduction in size of the midgut (mg) and the strong vascularization of the surrounding great blood vessels (bv, asterisks) after maturation. **(G, J)** Atokous midguts lie above the ventral longitudinal muscles (vlm) and transversal muscles (tm). **(H, K)** Female and **(I, L)** male epitokous midguts in **(G–I)** anterior and **(J–L)** posterior located segments of the same worm. The uniformly shaped atokous midguts are connected with suspension muscles (sm) of the dissepiments (arrowheads) to the body wall. Female guts are more compressed between the dissepiments after maturation than male guts (compare H to I as well as K to L) and therefore the supply with blood vessels (bv, asterisks) also becomes clearer. **(M–R)** Higher magnification SEM reveals visceral remodeling during maturation. **(M, P)** Atokous visceral muscles and **(N, Q)** female and **(O, R)** male epitokous visceral muscles in **(M–O)** anterior and **(P–R)** posterior located segments of the same worm. **(M, P)** While the atokous midgut display uniformly shaped visceral muscles covered with a thin coelothel, **(N, O, Q, R)** the epitokous visceral muscles appear to lose most of their covering. Rectangles show enlargements for Fig. 7. Abbr.: bv blood vessels, lvm longitudinal visceral muscles, mg midgut, sm suspension muscles, tm transversal muscles and vlm ventral longitudinal muscles. Scale bars: A–F 200 µm; G, J 500 µm; H, I, K, L 200 µm; M–R 50 µm.

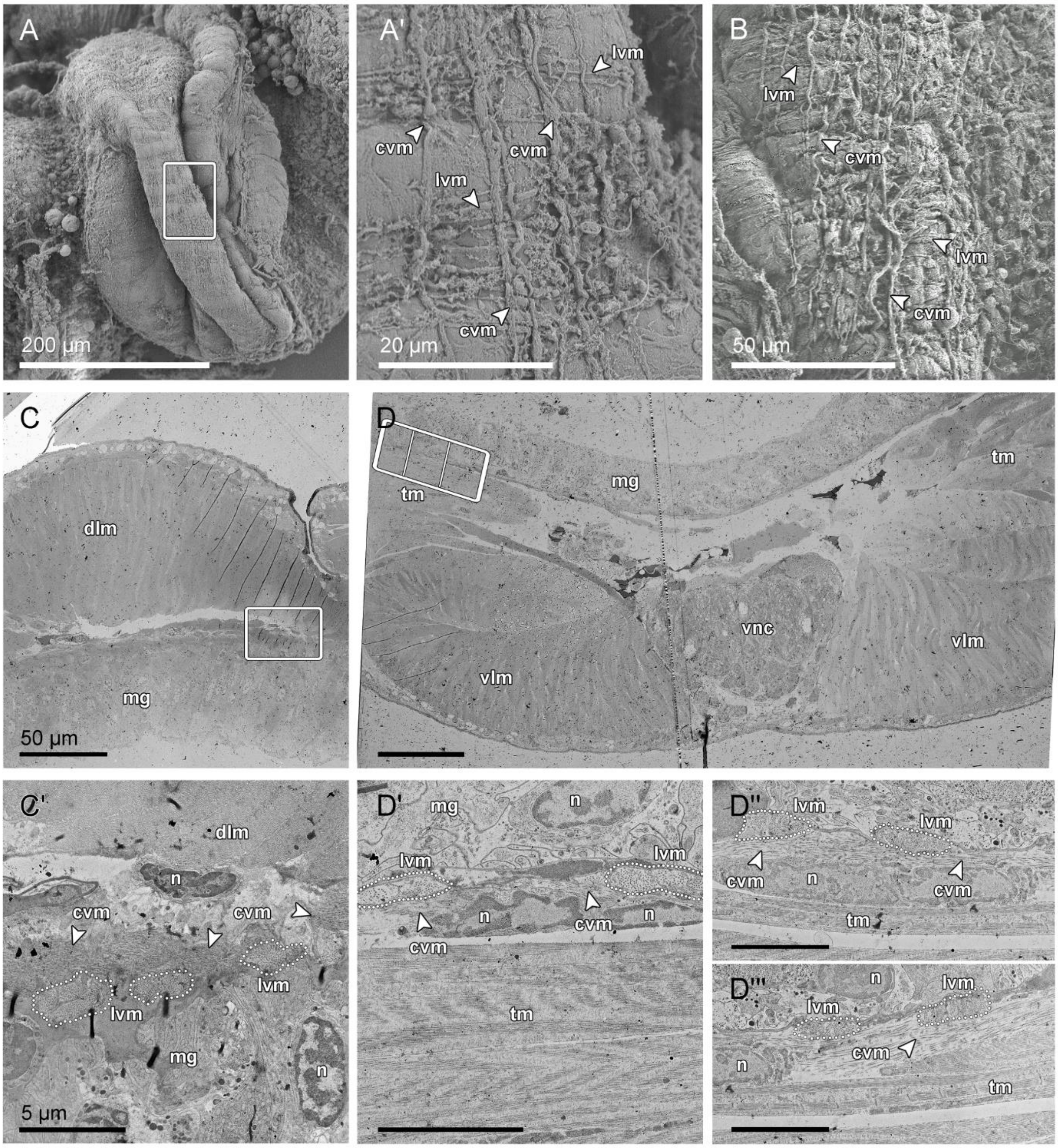

Figure S4: Structure and ultrastructure of *Platynereis* visceral muscles. (A, A´, B) Scanning electron micrographs of two epitokous midguts show the enlarged midgut region in **(A´)** and an additional midgut enlargement **(B)** with external circular visceral muscles (cvm) and internal longitudinal visceral muscles (lvm) with degraded coelothelial coverage. **(C, D)** The transmission electron micrographs show the overviews of the dorsal and ventral cross-sections for the enlarged regions (C´, D´, D´´, D´´´) in rectangles. **(C´, D´, D´´, D´´´)** The atokous visceral musculature consists of the

inner longitudinal visceral muscle fibers (lvm, dotted lines) that appear bundled and the overlying circular visceral muscle fibers (cvm, arrowheads), which together with the outer coelothel cells surround the midgut (mg). Abbr.: cvm circular visceral muscles, dlm dorsal longitudinal muscles, lvm longitudinal visceral muscles, mg midgut, n nuclei, tm transversal muscles, vlm ventral longitudinal muscles, vnc ventral nerve cord. Scale bars = A 200 µm; A´ 20 µm; B 50 µm; C, D 50 µm; C´, D´–D´´´ 5 µm.
