# Supplementary material for: Remodeling of the Platynereis Musculature during Sexual Maturation
Source: Biology (Basel). 2023 Feb 6;12(2):254. doi: 10.3390/biology12020254 (PMC9953025; doi:10.3390/biology12020254)
Supplement: Supplementary file 1 [file biology-12-00254-s001.zip › 6 biology-2147099 Supp. Data Sheet.pdf]

ratio calculation - % total area vs longitudinal muscle area

atok		total segment area [µm^2]		total dorsal longitudinal muscle (dlm) area [µm^2]		% relation total segment area vs total dlm	total ventral longitudinal muscle (vlm) area [µm^2]		% relation total segment area vs total vlm
sample	segment	total mean	standard deviation	total mean	standard deviation		total mean	standard deviation	
1	7-14	1174503	48762	52200	6582	4,44	47859	3701	4,07
	31-38	861892	41846	36651	5123	4,25	40893	2745	4,74
2	7-14	1318672	187435	84128	13246	6,38	55047	4934	4,17
	31-38	1142946	129988	75713	10849	6,62	60406	5853	5,29
3	7-14	673939	16782	35180	3475	5,22	31028	2072	4,60
	31-38	515599	39763	25926	4296	5,03	23347	4572	4,53
4	7-14	1433884	46099	42996	4824	3,00	48949	5753	3,41
	31-38	1364230	46108	46688	3871	3,42	46997	4912	3,44
5	7-14	1580660	50652	84584	10518	5,35	60246	6206	3,81
	31-38	1013707	45246	44010	8708	4,34	46026	9047	4,54
6	7-14	1513105	56368	75590	4500	5,00	50454	2500	3,33
	31-38	633877	39455	21995	1657	3,47	24546	2650	3,87
7	7-14	1850524	188245	92241	8681	4,98	65855	4517	3,56
	31-38	1565904	73193	65944	10826	4,21	52719	3690	3,37

	segment	total mean	standard deviation	total mean	standard deviation	mean % relation	standard deviation	total mean	standard deviation	mean % relation	standard deviation
	7-14	1363612	370790	66703	22806	4,91	1,03	51348	11067	3,85	0,46
	31-38	1014022	378576	45275	19782	4,48	1,09	42133	13836	4,25	0,71

ratio calculation - % total area vs longitudinal muscle area

female		total segment area [µm^2]		total dorsal longitudinal muscle (dlm) area [µm^2]		% relation total segment area vs total dlm	total ventral longitudinal muscle (vlm) area [µm^2]		% relation total segment area vs total vlm
sample	segment	total mean	standard deviation	total mean	standard deviation		total mean	standard deviation	
1	7-14	1109317	34381	18192	1816	1,64	23819	3369	2,15
	31-38	830639	55082	24543	6273	2,95	26764	2437	3,22
2	7-14	1853722	76026	16077	1764	0,87	19148	3786	1,03
	31-38	1496426	34282	25486	1863	1,70	25853	1269	1,73
3	7-14	2265208	67304	15244	2308	0,67	19077	3210	0,84
	31-38	1748065	70106	16460	2681	0,94	22089	3418	1,26
4	7-14	3029957	82560	22728	4361	0,75	21633	3177	0,71
	31-38	1510869	178904	25787	4457	1,71	35606	4417	2,36
5	7-14	1486409	112459	11513	1911	0,77	14532	2714	0,98
	31-38	1118266	68323	16887	2022	1,51	18616	1782	1,66
6	7-14	2005352	68200	21679	4070	1,08	26583	3164	1,33
	31-38	2008027	38049	23727	3224	1,18	21116	4851	1,05
7	7-14	1491610	86486	9355	2623	0,63	11075	1307	0,74
	31-38	1196085	50646	15038	2576	1,26	18894	2700	1,58
8	7-14	1587377	86045	17145	5047	1,08	15145	2979	0,95
	31-38	1339577	33915	18828	3358	1,41	19327	2147	1,44

	segment	total mean	standard deviation	total mean	standard deviation	mean % relation	standard deviation	total mean	standard deviation	mean % relation	standard deviation
	7-14	1853619	593653	16492	4576	0,94	0,33	18877	5143	1,09	0,47
	31-38	1405994	370507	20844	4482	1,58	0,61	23533	5768	1,79	0,69

ratio calculation - % total area vs longitudinal muscle area

male		total segment area [μm²]		total dorsal longitudinal muscle (dlm) area [μm²]		% relation total segment area vs total dlm	total ventral longitudinal muscle (vlm) area [μm²]		% relation total segment area vs total vlm
sample	segment	total mean	standard deviation	total mean	standard deviation		total mean	standard deviation	
1	7-14	1361662	117623	13751	2842	1,01	13435	1928	0,99
	31-38	1669426	42241	30370	2234	1,82	24215	1373	1,45
2	7-14	1422861	58894	10317	2210	0,73	9803	1191	0,69
	31-38	1294621	79105	27279	2543	2,11	27930	2097	2,16
3	7-14	1595780	121114	18876	3508	1,18	21898	4189	1,37
	31-38	1147339	84962	36244	1831	3,16	32728	2602	2,85
4	7-14	2389050	118873	32849	4793	1,37	31754	2838	1,33
	31-38	1577668	135051	45711	5322	2,90	40247	3080	2,55
5	7-14	1810142	127568	52506	6499	2,90	37823	4131	2,09
	31-38	1381647	61907	39608	4709	2,87	36857	6622	2,67
6	7-14	1584326	83065	40973	6232	2,59	37578	1538	2,37
	31-38	1322660	44577	35987	2227	2,72	26242	2415	1,98
7	7-14	1441977	158505	25588	3028	1,77	24969	1648	1,73
	31-38	1589705	144756	42557	2883	2,68	33806	2500	2,13
8	7-14	2075680	153607	25348	4694	1,22	21178	3599	1,02
	31-38	1408578	75716	34431	5009	2,44	28871	2211	2,05

	segment	total mean	standard deviation	total mean	standard deviation	mean % relation	standard deviation	total mean	standard deviation	mean % relation	standard deviation
	7-14	1710185	360421	27526	14154	1,60	0,77	24805	10399	1,45	0,58
	31-38	1423955	176023	36523	6076	2,59	0,44	31362	5507	2,23	0,45
