# Supplementary figures and images for: Remodeling of the Platynereis Musculature during Sexual Maturation
Source: Biology (Basel). 2023 Feb 6;12(2):254. doi: 10.3390/biology12020254 (PMC9953025; doi:10.3390/biology12020254)

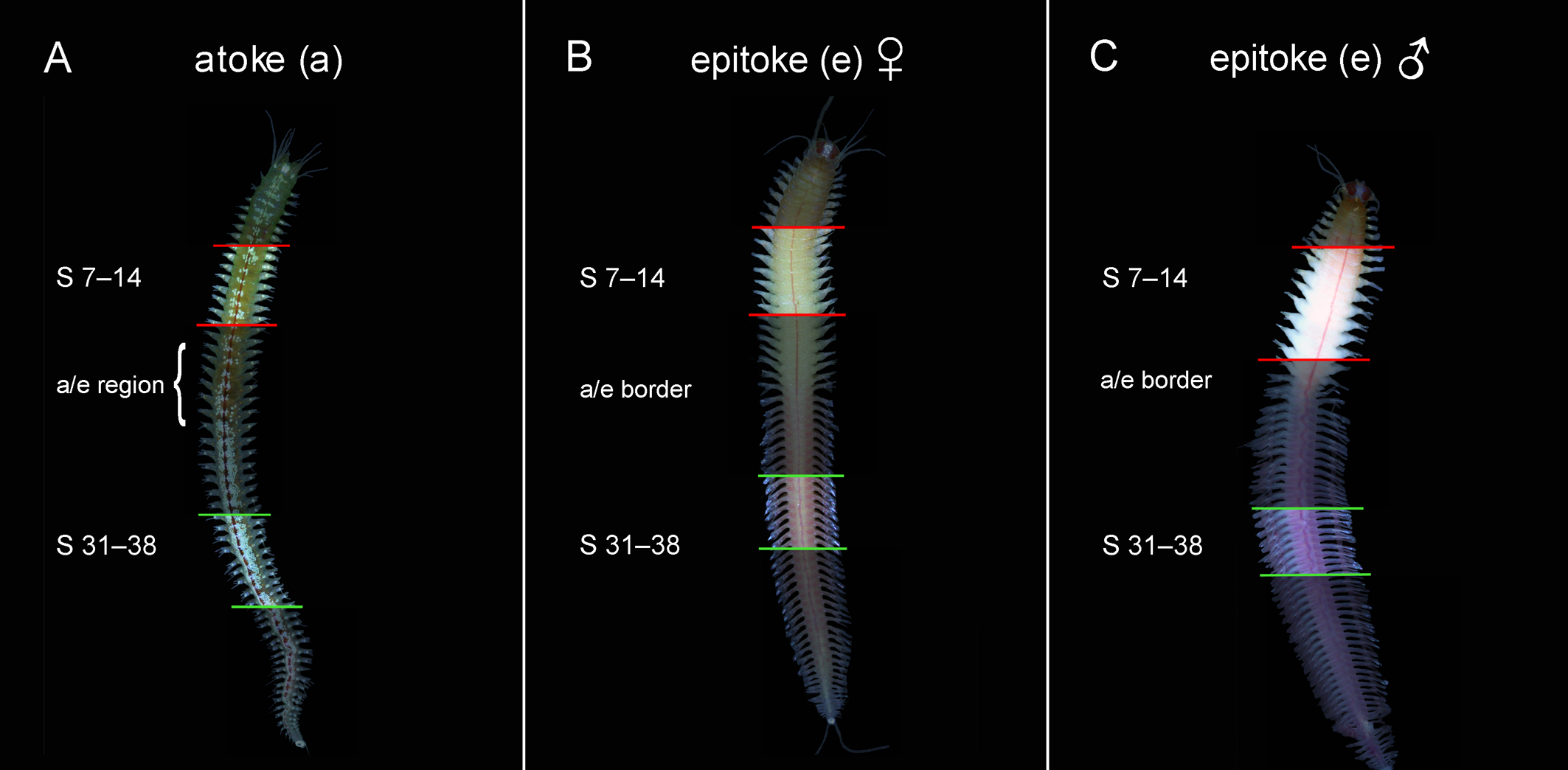

Supplement: Supplementary file 1 [file biology-12-00254-s001.zip › 1 Figure S1 300 dpi.tif]

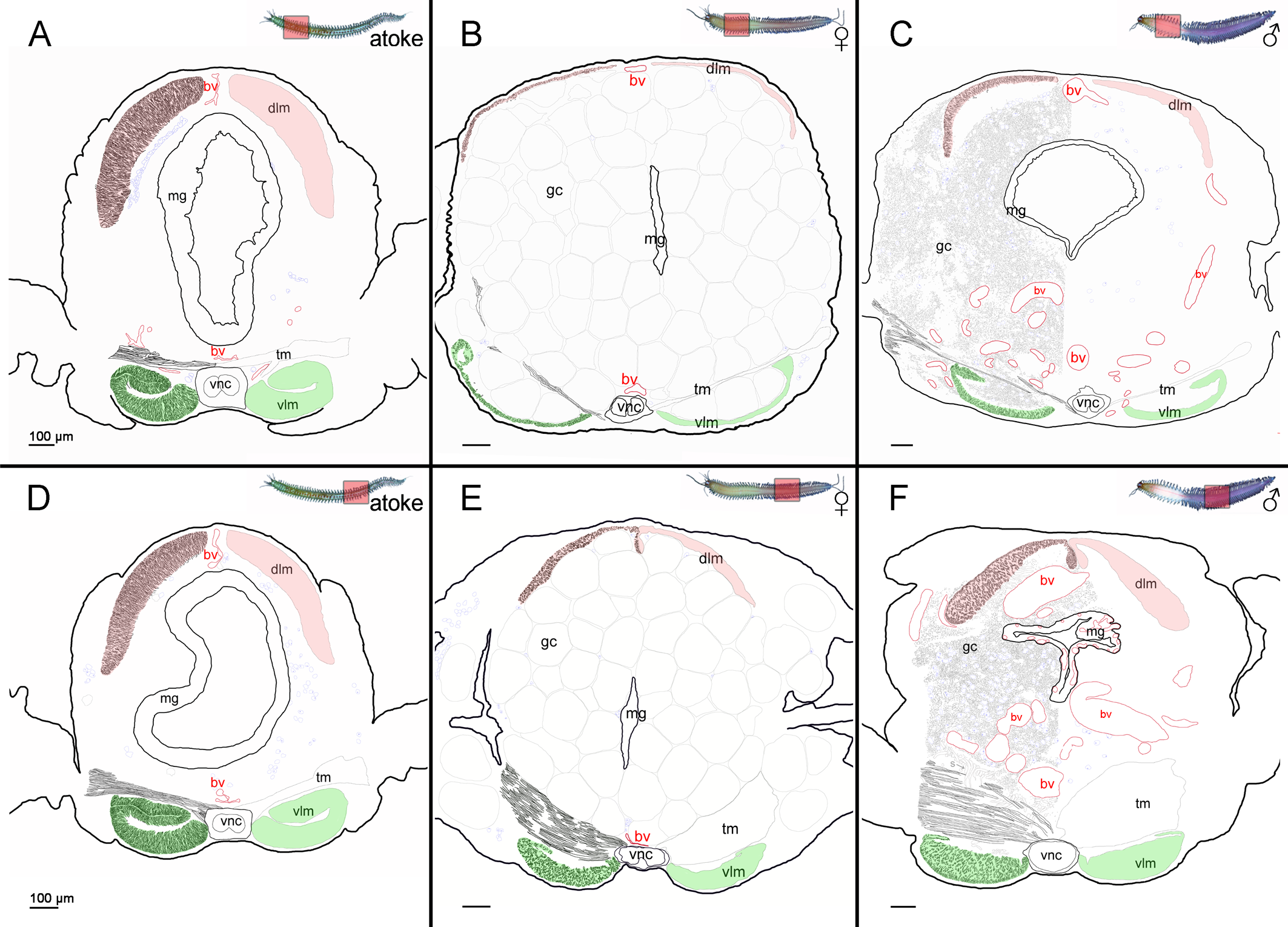

Supplement: Supplementary file 1 [file biology-12-00254-s001.zip › 2 Figure S2 300 dpi.tif]

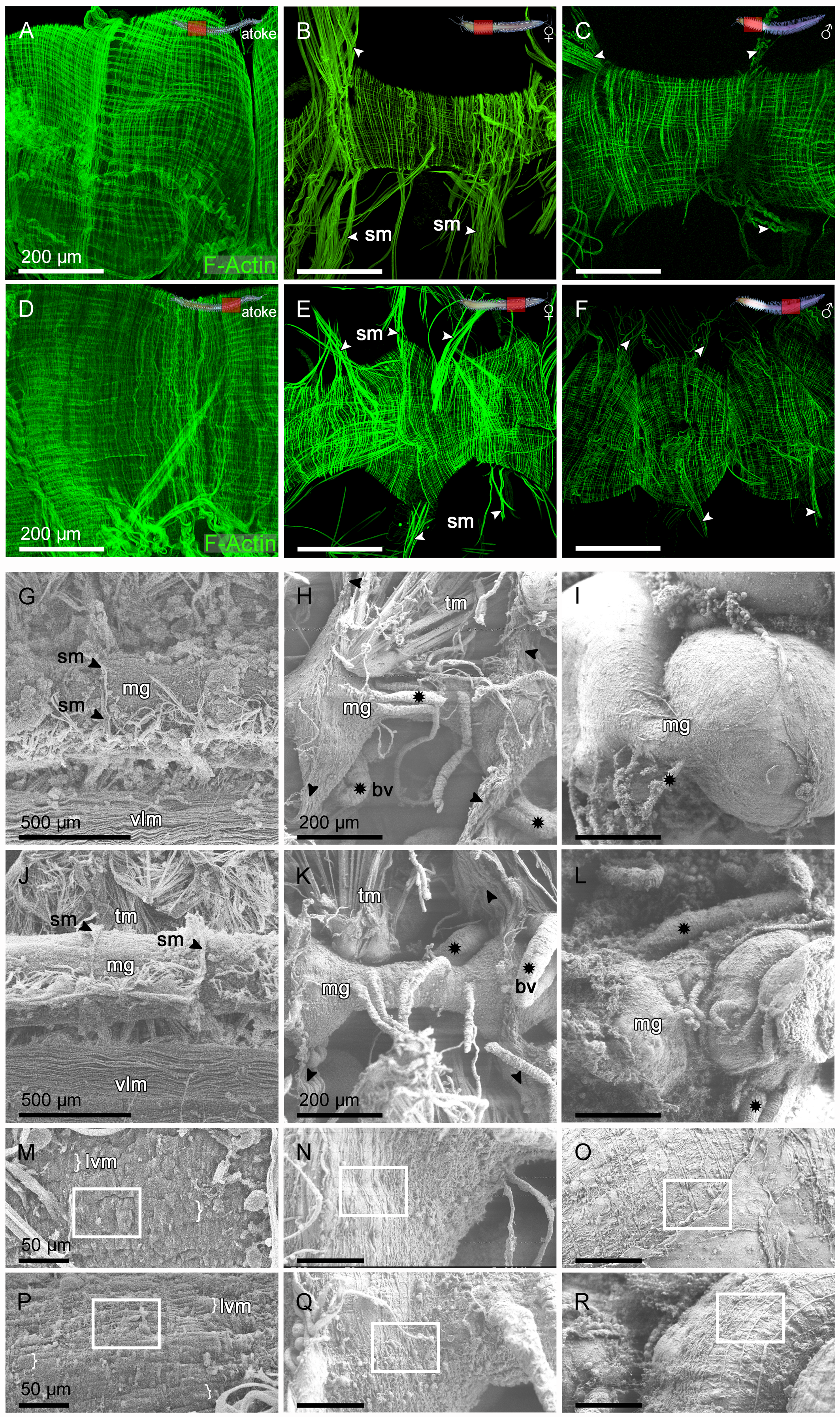

Supplement: Supplementary file 1 [file biology-12-00254-s001.zip › 3 Figure S3 300 dpi.tif]

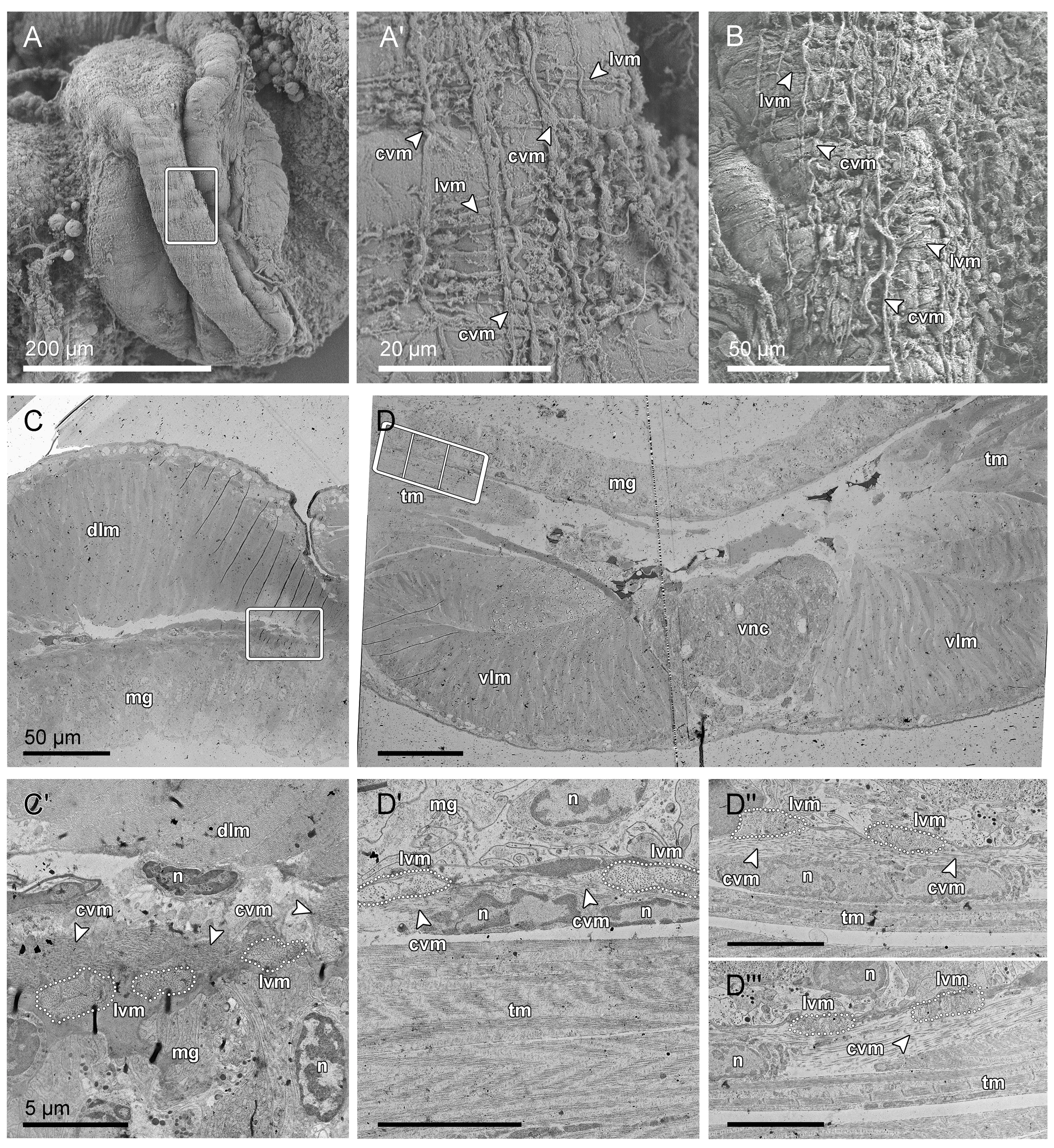

Supplement: Supplementary file 1 [file biology-12-00254-s001.zip › 4 Figure S4 300 dpi.tif]
